# Development of a deep learning model for automatic detection of narrowed intervertebral disc space sites in caudal thoracic and lumbar lateral X-ray images of dogs

**DOI:** 10.3389/fvets.2024.1453765

**Published:** 2024-11-27

**Authors:** Junseol Park, Hyunwoo Cho, Yewon Ji, Kichang Lee, Hakyoung Yoon

**Affiliations:** ^1^Department of Veterinary Medical Imaging, College of Veterinary Medicine, Jeonbuk National University, Iksan, Republic of Korea; ^2^College of Veterinary Medicine, Biosafety Research Institute, Jeonbuk National University, Iksan, Republic of Korea; ^3^Department of Electronic Engineering, Sogang University, Seoul, Republic of Korea

**Keywords:** intervertebral disc disease, artificial intelligence, disc space, segmentation, detection, canine

## Abstract

Intervertebral disc disease is the most common spinal cord-related disease in dogs, caused by disc material protrusion or extrusion that compresses the spinal cord, leading to clinical symptoms. Diagnosis involves identifying radiographic signs such as intervertebral disc space narrowing, increased opacity of the intervertebral foramen, spondylosis deformans, and magnetic resonance imaging findings like spinal cord compression and lesions, alongside clinical symptoms and neurological examination findings. Intervertebral disc space narrowing on radiographs is the most common finding in intervertebral disc extrusion. This study aimed to develop a deep learning model to automatically recognize narrowed intervertebral disc space on caudal thoracic and lumbar X-ray images of dogs. In total, 241 caudal thoracic and lumbar lateral X-ray images from 142 dogs were used to develop and evaluate the model, which quantified intervertebral disc space distance and detected narrowing using a large-kernel one-dimensional convolutional neural network. When comparing veterinary clinicians and the deep learning model, the kappa value was 0.780, with 81.5% sensitivity and 95.6% specificity, showing substantial agreement. In conclusion, the deep learning model developed in this study, automatically and accurately quantified intervertebral disc space distance and detected narrowed sites in dogs, aiding in the initial screening of intervertebral disc disease and lesion localization.

## 1 Introduction

Intervertebral disc disease (IVDD) is the most common cause of neurologic dysfunction in dogs, encompassing various lesions affecting the intervertebral disc space (IVDs) (1–3). Since IVDD was first described in dogs in 1896, the terminology and classification have evolved with advances in research and imaging diagnostic techniques (2, 3). Until the late 1900s, X-ray was the most widely used diagnostic tool for IVDD in dogs. However, subsequent studies have shown that magnetic resonance imaging (MRI) is necessary to confirm herniated disc material and spinal cord compression (2, 4). Therefore, the current gold standard for diagnosing IVDD involves a combination of physical and neurological examinations, as well as radiographic and MRI findings (1, 5–9). Although MRI scans are helpful for the accurate diagnosis of IVDD, in veterinary practice, this can be impractical due to the long duration of anesthesia needed for MRI scans (4).

IVDD is a widely used term that includes a variety of diseases affecting the intervertebral discs, but can be broadly categorized into disc extrusion and protrusion (3). Radiologic findings in disc extrusion include IVDs narrowing (70%) and disc mineralization (42 percent), while those in disc protrusion include sclerosis of the vertebral body endplates (67%), spondylosis deformans (47%), and IVDs narrowing (25%) (2, 10). Of these various radiologic findings, IVDs narrowing is common in both disc extrusion and protrusion and this means that detecting IVDs narrowing on radiography may be the first step in diagnosing IVDD (10).

While a negative result (the narrowed IVDs is not visible on the radiograph) does not rule out IVDD, a positive result provides valuable clinical information. In some cases, radiographic imaging allows for a tentative diagnosis of IVDD, and in others, the next diagnostic step, MRI (including myelography) may be required (3). Therefore, in all cases where IVDD is suspected based on clinical symptoms, radiography is a diagnostic imaging modality that cannot be omitted from the proper diagnostic process.

However, due to the complexity and interobserver variability in diagnosing IVDD, in human medicine, deep learning models, a form of artificial intelligence, have been applied to automatically detect diseases for the objective and accurate diagnosis of various disc conditions (11–15). Despite the various deep learning approaches have been proposed for the automatic analysis of medical images, conventional detection and classification models are not suitable for accurately quantifying IVDs or detecting narrowed intervertebral disc regions (16, 17). Because the IVDs is a very small region, conventional segmentation approaches require very high accuracy, which is often impractical and results in inaccurate detection of narrowed IVDs. Furthermore, determining the narrowed IVDs requires comparison with other IVDs, necessitating that the deep learning model considers the global context.

Therefore, this study aimed to develop a new model to automatically detect narrowed IVDs sites by utilizing a vertebral body segmentation model to quantify the caudal thoracic and lumbar IVDs on a pixel basis and to design a large-kernel one-dimensional convolutional neural network (1D-CNN) to automatically detect narrowed IVDs sites, unlike the common approach of segmenting the IVDs directly.

## 2 Materials and methods

### 2.1 Patient dataset

This was a retrospective study, and patients who visited the Jeonbuk National University Animal Medical Center between April 2017 and October 2023 and underwent caudal thoracic and lumbar vertebrae X-rays were selected. In 142 dogs, X-ray images (ECO-BT-525 VET, EcoRay, Gwangju, Korea) were obtained and used to develop the deep learning models. Caudal thoracic or lumbar MRI (Vet-MR Grande, Esaote, Genova, Italy) was performed under general respiratory anesthesia in 30 of the 142 dogs. Dogs without specific clinical signs related to disc disease and patients with suspected disc disease based on physical and neurological examinations, such as spinal pain, proprioceptive ataxia, paresis, and plegia, were also included in the study. This study was approved by the Institutional Animal Care and Use Committee of Jeonbuk National University (approval no. JBNU NON2023-023).

### 2.2 Image dataset

#### 2.2.1 Radiographic image acquisition

A total of 241 right lateral X-ray images (ECO-BT-525 VET; EcoRay, Gwangju, Korea) from 142 dogs were used to develop a narrowed IVDs site detection model. For the dataset, images acquired under conditions of 66–70 kVp and 2.6–3.0 mAs were used. For caudal thoracic lateral X-ray images, the center of the beam was located at approximately T12-T13, with the field of view (FOV) spanning from T8 to L4, allowing for variations between dogs. For lumbar lateral X-ray images, the beam center was at L3-L4, covering the images from T12 to the cranial level of the caudal vertebrae. The focal spot to detector distance (FDD) was fixed at 80 cm, all images were obtained with the vertebrae as straight as possible and post-processed to maintain adequate contrast. The dataset was divided into training and validation sets in an approximate 80:20 ratio, with random selection for training. Specifically, 106 caudal thoracic lateral X-ray images (85 for training and 21 for validation dataset) and 135 lumbar lateral X-ray images (107 for training and 28 for validation dataset) were used in this study. No suspicious lesions of vertebral disease, such as vertebral tumors and Schmorl’s nodes, were observed in the acquired X-ray images, which could affect the results of this study. Images containing mismatched vertebral endplates, motion artifacts, and vertebral rotation in the acquired X-ray images were excluded from the study.

#### 2.2.2 Evaluation of radiographic images

Three veterinary clinicians analyzed the X-ray images and diagnosed the areas that they commonly judge to be narrow as narrowed IVDs sites. In the caudal thoracic lateral X-ray images, the IVDs from T10 to L3 was evaluated, and in the lumbar lateral X-ray images, the IVDs from L1 to L7 was evaluated. In addition, for each individual IVDs, checked how many narrowed IVDs were found.

#### 2.2.3 MR image acquisition for comparison with radiographic evaluation

Of the 142 dogs, 30 underwent MRI scans using a 0.25 Tesla MRI machine (Vet-MR Grande, Esaote, Geneva, Italy). A total of 19 dogs underwent caudal thoracic MRI, 15 dogs underwent lumbar MRI and 4 dogs underwent caudal thoracic and lumbar MRI both. Of the dogs included in the validation dataset, nine underwent MRI, and the images of five caudal thoracic and four lumbar MRI scans were analyzed to assess the performance of deep learning model. The patients were maintained in dorsal recumbency during the MRI scans, with the vertebral body as straight as possible.

#### 2.2.4 Analysis on MR images

Median plane T1-weighted images [slice thickness: 3.0 or 3.5 mm, repetition time (TR) = 520–770 ms, echo time (TE) = 26 ms] and median plane T2-weighted images (slice thickness: 3.0 or 3.5 mm, TR = 1,360–2,700 ms, TE = 100 or 120 ms) were used to identify narrowed IVDs sites. To determine the presence of spinal cord compression, median plane T1 and T2-weighted images were referenced, whereas transverse plane T1-weighted images (slice thickness: 2.5–6.0 mm, TR = 600 ms, TE = 26 ms) and transverse plane T2-weighted images (slice thickness: 3.0–5.0 mm, TR = 2,800–4,840 ms, TE = 80 ms) were evaluated to calculate the compression ratio. The spinal cord compression ratio was defined as the ratio of the cross-sectional area (CSA) of the maximally compressed spinal cord parenchyma to the CSA of the normal spinal cord parenchyma (16). Spinal cord compression was considered present if a reduction in CSA of 25% or more was identified compared to the CSA of the normal spinal cord parenchyma, indicating moderate to severe compression (2, 18). To determine areas of IVDs narrowing or spinal cord compression on MR images, two veterinarians analyzed the images and selected the areas they agreed upon. These areas were then compared with IVDs identified as narrow on caudal thoracic and lumbar lateral X-ray images. In addition, for each individual IVDs, checked how many spinal cord compression were found and assessed the correspondence with the radiologically observed IVDs narrowing. Additionally, the T10-T11 and L5-L6 regions, which are commonly narrowed on X-rays in most dogs (19), were excluded for additional comparison with MR images.

### 2.3 Deep learning model development

#### 2.3.1 Manual segmentation

**Figure 1:**
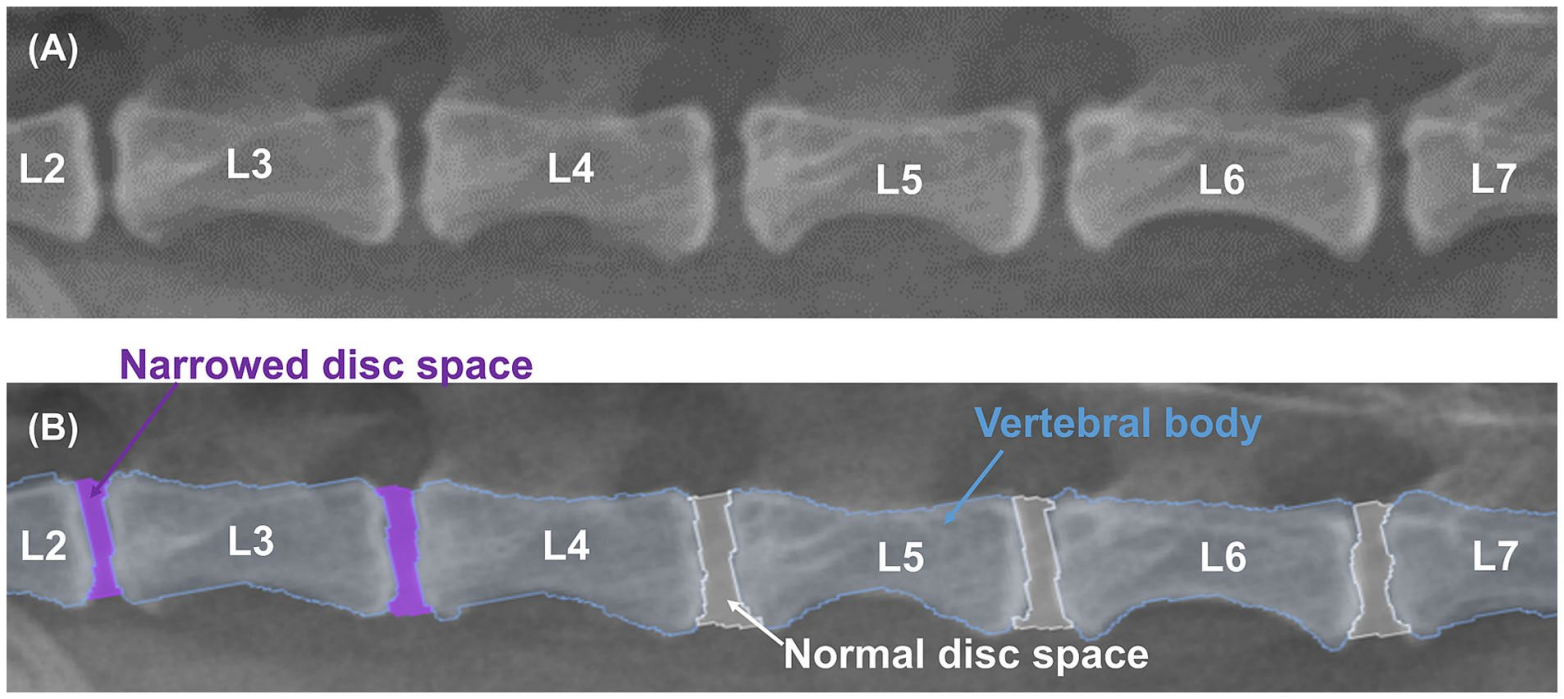
Example of manual segmentations. Lateral lumbar X-ray images **(A)** show the vertebral body (sky blue), normal IVDs (white), and narrowed IVDs (purple), which were labeled with separate colors using a segmentation tool (MediLabel software) for differentiation **(B)**.

X-ray images used in this study were labeled by 13 veterinary clinicians (residents in the Veterinary Medical Imaging Department of the Teaching Hospital of Jeonbuk National University) using MediLabel software (Ingradient, Inc., Seoul, South Korea). In the caudal thoracic and lumbar X-ray images, separate colors were used for labeling to distinguish the vertebral body from the normal or narrowed IVDs. Figure 1 shows an example of manual segmentation of a lumbar X-ray image.

#### 2.3.2 Preprocessing algorithm to quantify IVDs

**Figure 2:**
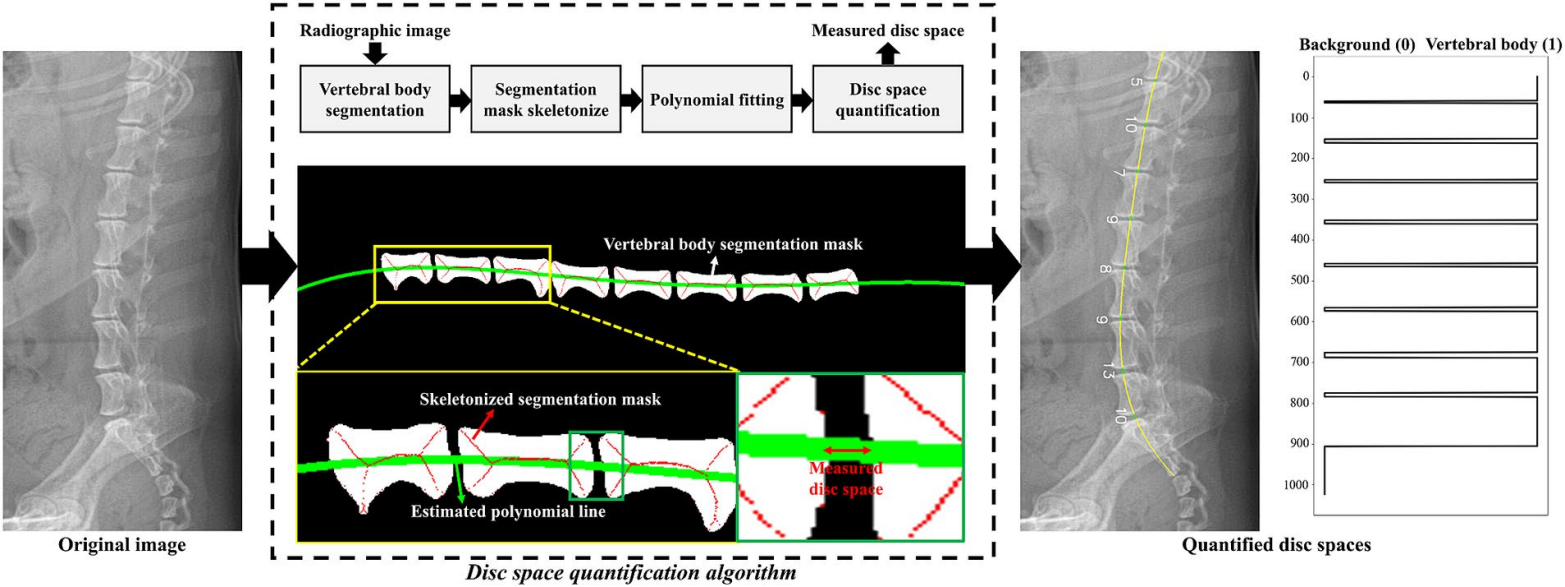
Graphical illustration of the proposed IVDs quantification algorithm. The green line indicates the estimated polynomial line passing through the centers of the vertebral bodies. Using this polynomial line, a vector of length 1,024 was generated based on the index values from the segmentation result. In the quantified vector, values of 0 and 1 denoted the background and vertebral body, respectively. IVDs were measured by counting the pixels between each vertebral body. An example of the quantified vector is plotted on the right side of the figure.

Figure 2 illustrates the proposed preprocessing algorithm for quantifying IVDs from radiographic images. The algorithm began with radiographic images and segmentation results of the vertebral bodies, which were obtained using various segmentation methods. The radiographic images and segmentation results were resized to a height of 1,024 pixels and a width of 512 pixels to ensure a uniform data shape. The algorithm estimated a curved line connecting the centers of the vertebral bodies to quantitatively measure the IVDs between them. As shown in the flowchart in Figure 2, each segmented vertebral body was skeletonized using morphological operations to determine the shape-independent center of the vertebral body (20). A 4th-order polynomial curve was then estimated by minimizing the squared error of the skeletonized vertebral bodies. Using this preprocessing algorithm, radiographic images were transformed into quantified IVDs, represented as a vector of length 1,024. Each component of the quantified vector was classified into three categories (no IVDs, normal IVDs, or narrowed IVDs) using a large-kernel 1D-CNN, as described in the following section.

#### 2.3.3 Model architecture to detect narrowed IVDs

**Figure 3:**
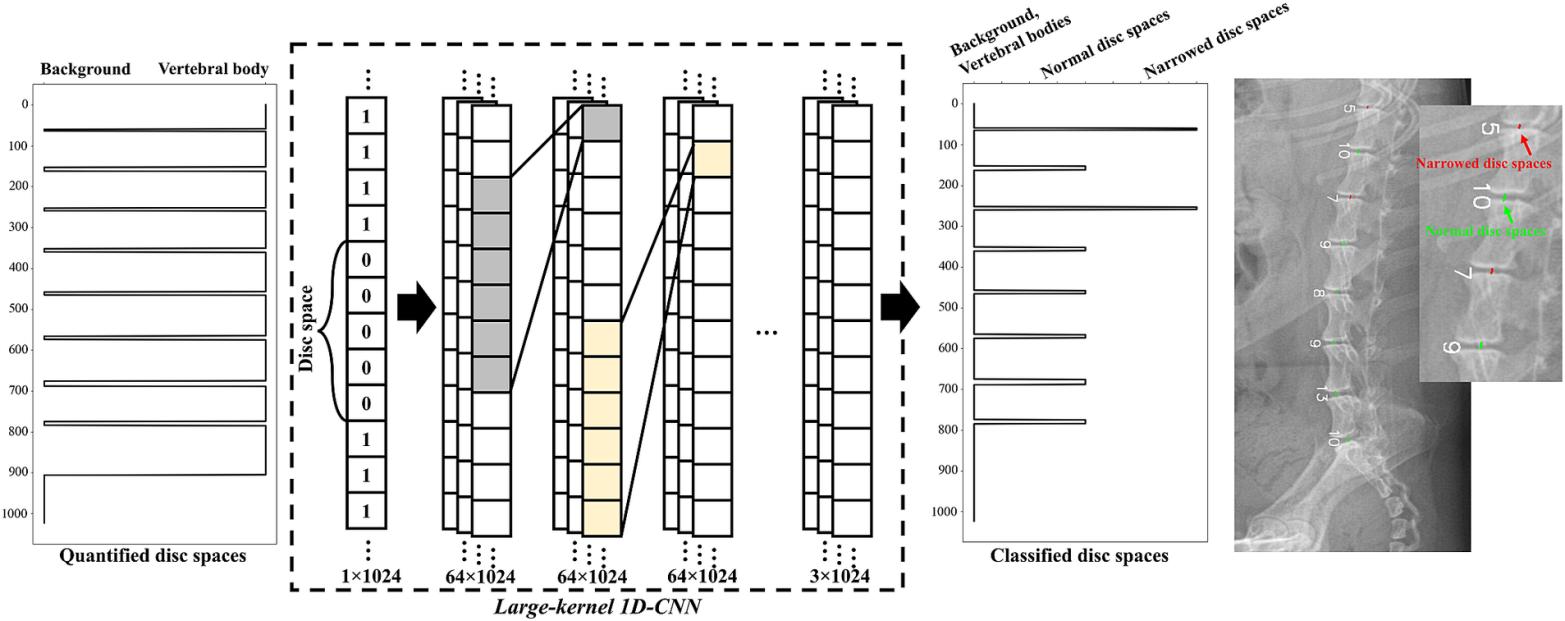
Schematic representation of the large-kernel one-dimensional convolutional neural network (1D-CNN) model. The quantified IVDs (a vector of length 1,024) was fed into the network. Each intermediate layer was followed by 1D convolution layers with a kernel size of 151 and 64 channels. The output of the model consisted of three categories: no IVDs, normal IVDs, and narrowed IVDs. An example of the classified IVDs is presented on the right side of the figure.

Figure 3 shows a schematic of the designed large-kernel 1D-CNN model. To compare each IVDs with adjacent IVDs, the model was designed with a large receptive field achieved by repeated 1D convolution operations with a kernel size of 151. The number of channels was set to 64, and the stride was set to 1 to retain dimensionality. Five intermediate 1D convolution layers were used before the classification layer. For the intermediate layers, batch normalization (21) and residual connections (22) were utilized to stabilize the model. The kernel size, number of channels, and stride parameters were empirically determined in our experiments.

The designed large-kernel 1D-CNN model was trained by minimizing a combined loss function comprising cross-entropy loss and focal loss (23). Focal loss was utilized to mitigate the class imbalance between normal and narrowed IVDs. The combined loss function used in this study was formulated as follows:


$$L_{c}^{\left(c\right)}=-y_{\text{true}}^{\left(c\right)}\log\left(y_{\text{pred}}^{\left(c\right)}\right)$$

$$L_{\text{combined}}=\sum_{c=1}^{C}\left[\alpha_{c}{\left(1-y_{\text{pred}}^{\left(c\right)}\right)}^{\gamma }\cdot L_{c}^{\left(c\right)}+L_{c}^{\left(c\right)}\right]$$

where C is the number of classes, $y_{\text{true}}^{\left(c\right)}$ is the true label for the $c_{th}$ class, $y_{\text{pred}}^{\left(c\right)}$ is the predicted probability for the $c_{th}$ class, $\alpha_{c}$ is the balancing factor, and γ is the focusing factor. In this study, $\alpha_{c}$ and γ were set to 0.25 and 2.0, respectively. The loss function was minimized by Adam optimizer (24) with a learning rate of $1e^{-5}$.

### 2.4 Time measurement for evaluation of narrowed IVDs

For the 49 lateral caudal thoracic and lumbar X-ray images used in validation, the time required per image for detecting narrowed IVDs by a veterinary clinician and the deep learning model was recorded and compared.

### 2.5 Model accuracy and statistical analysis

Fleiss’ kappa analysis was performed to determine the agreement among three veterinary clinicians regarding the narrowed IVDs sites. Cohen’s kappa analysis was used to evaluate the consistency between veterinary clinicians and the deep learning model in detecting narrowed IVDs sites in caudal thoracic and lumbar lateral X-ray images.

Cohen’s kappa analysis was also performed to assess inter-veterinary clinician agreement for areas of narrowed IVDs and spinal cord compression on MR images and to assess the correlation of lesions identified on MR images with areas judged by veterinary clinicians and the deep learning model as areas of IVDs narrowing on caudal thoracic and lumbar lateral X-ray images, respectively.

Kappa values were interpreted as follows: values ≤0.00–0.20 indicated non-to-slight, 0.21–0.40 indicated fair, 0.40–0.60 indicated moderate, 0.60–0.80 indicated substantial, and 0.80–1.00 indicated almost perfect agreement (25).

Based on the average IVDs distance values quantified by the deep learning model, the ratio of each IVDs distance was obtained and used in the analysis. To confirm the association between this ratio and the IVDs determined to be narrow by the veterinary clinician, receiver operating characteristic (ROC) curves and the area under the curve (AUC) with 95% confidence intervals (CIs) using the IVDs considered to be narrow by veterinary clinicians as the classifier was generated. Sensitivity and specificity for the calculated ratio of IVDs distance were determined with the Youden index. AUC values were interpreted as follows: 0.5–0.59 indicated unsatisfactory, 0.6–0.69 indicated satisfactory, 0.7–0.79 indicated good, 0.8–0.89 indicated very good, and 0.9–1.0 indicated excellent classifier (26).

Statistical analysis was performed using SPSS version 29.0 (SPSS Corp., Armonk, NY, USA), and experimental values were considered significant at ^*^p < 0.05 or ^**^p < 0.01.

## 3 Results

### 3.1 Animals

The study included 76 male dogs (21 intact, 55 castrated) and 66 female dogs (19 intact, 47 spayed), spanning 27 breeds: Maltese (*n* = 36), Poodle (*n* = 15), Pomeranian (*n* = 14), Dachshund (*n* = 12), Mix (*n* = 10), Pekingese (*n* = 8), Shih Tzu (*n* = 8), Cocker Spaniel (*n* = 5), Miniature Poodle (*n* = 5), Chihuahua (*n* = 4), Bichon Frise (*n* = 3), Beagle (*n* = 3), German Shepherd (*n* = 2), Golden Retriever (*n* = 2), Old English Sheepdog (*n* = 2), Yorkshire Terrier (*n* = 2), Jindo (*n* = 1), Boston Terrier (*n* = 1), Labrador Retriever (*n* = 1), Miniature Pinscher (*n* = 1), Pompitz (*n* = 1), Samoyed (*n* = 1), Schnauzer (*n* = 1), Shetland Sheepdog (*n* = 1), Spitz (*n* = 1), Welsh Corgi (*n* = 1), and Whippet (*n* = 1). The weight range in the study was 114 individuals under 10 kg, 14 individuals between 10 and 20 kg, and 9 individuals over 20 kg and average weight was 7.16 kg (1.64–36 kg), and information regarding body weight was unavailable for five dogs. The average age was 8.36 years (range: 0.7–17 years).

### 3.2 T10-T11 IVDs is most frequently observed to be narrow, in normal conditions

**Figure 4:**
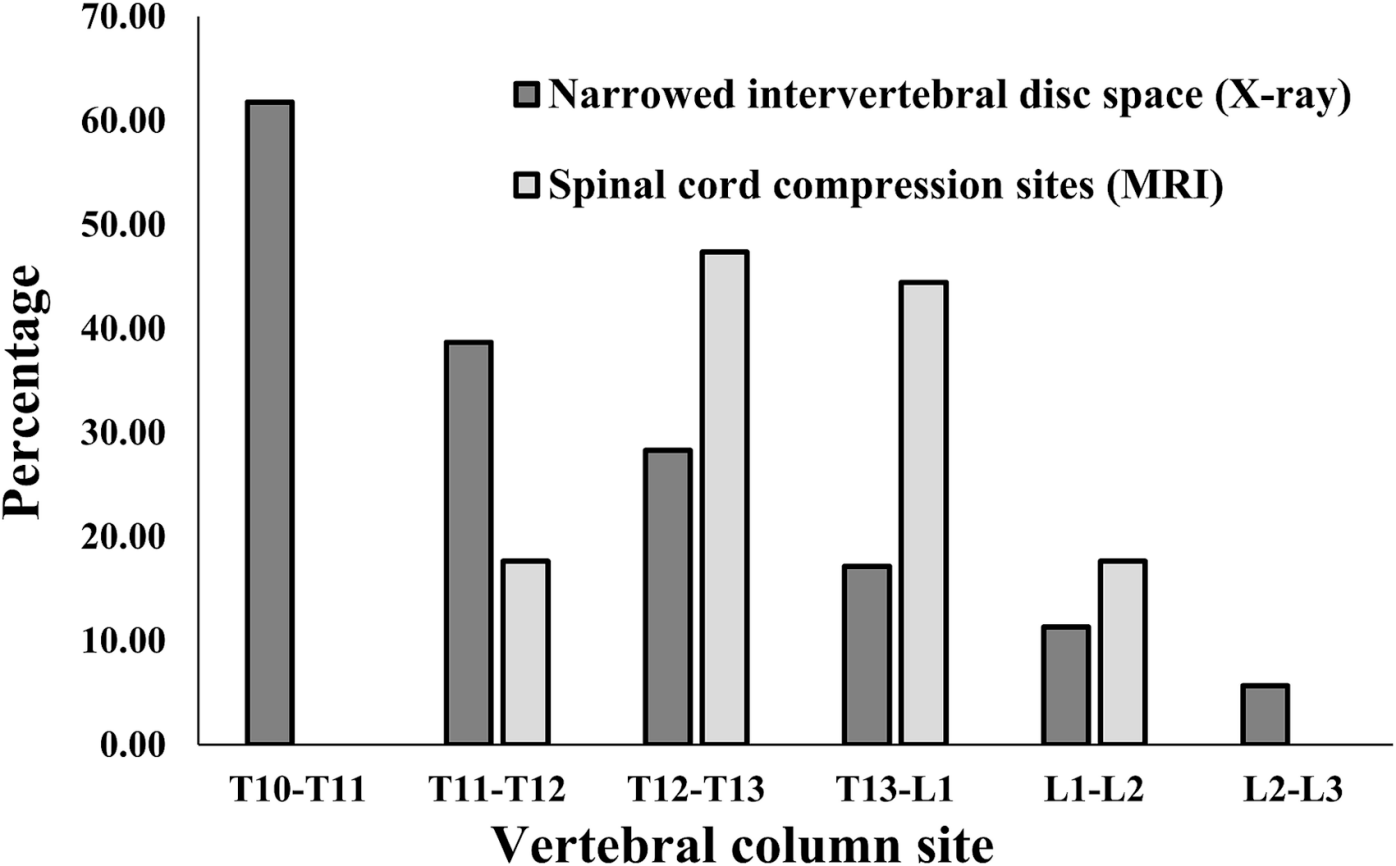
Distribution of sites for IVDs narrowing in 106 caudal thoracic lateral X-ray images and for spinal cord compression in 19 caudal thoracic MRI scan in total dataset. T, thoracic; L, lumbar.

**Figure 5:**
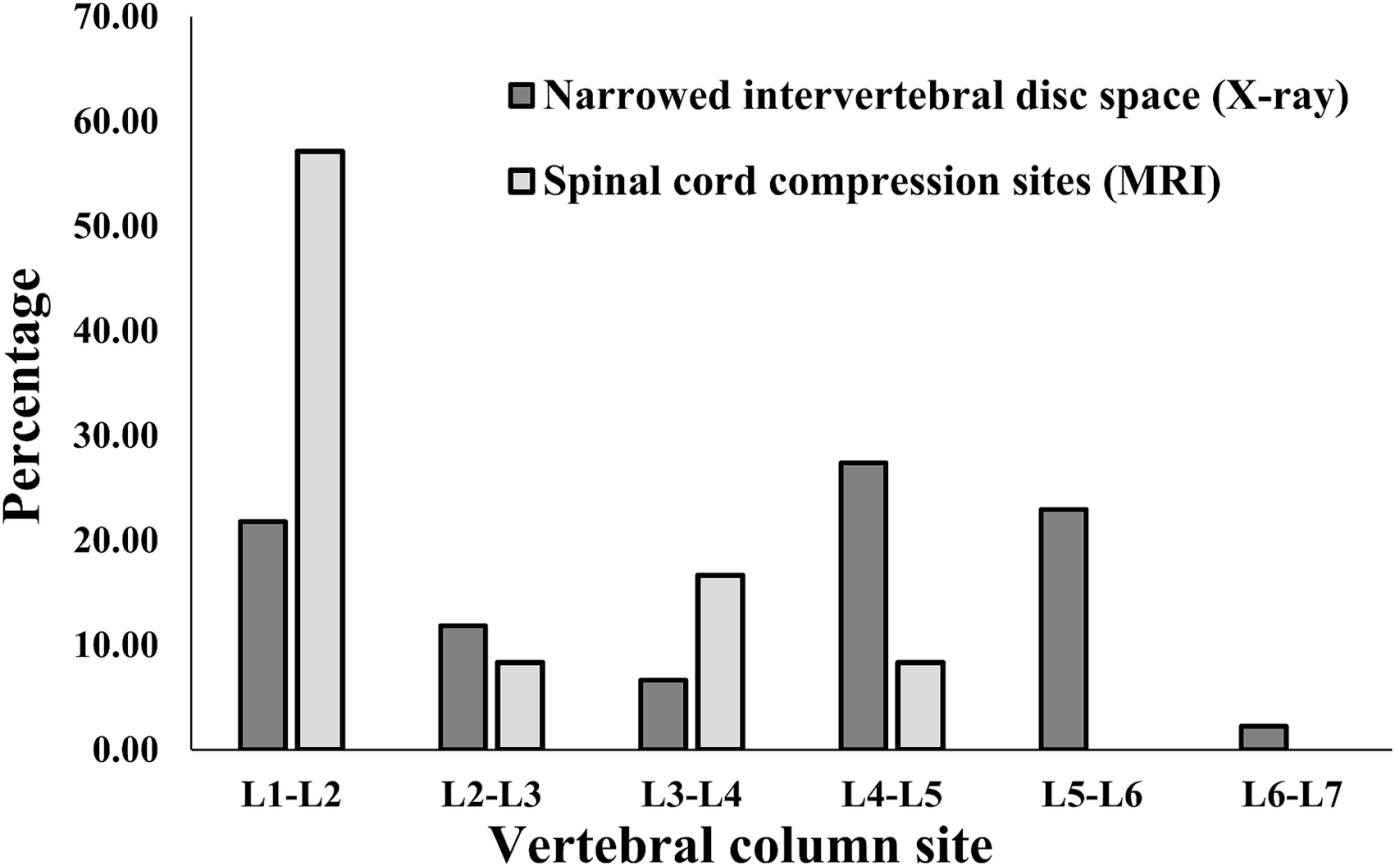
Distribution of sites for IVDs narrowing in 135 lumbar lateral X-ray images and for spinal cord compression in 15 caudal thoracic MRI scan in total data set. L, lumbar.

When checking how many narrowed IVDs were found in individual IVDs, among all caudal thoracic lateral X-ray images, T10-T11 was the most frequently identified as a narrowed IVDs with 61.8% (63/102), and among lumbar lateral X-ray images, the overall percentage was similar, but L4-L5 was identified as a narrowed IVDs more frequently with 27.4% (37/135) and L5-L6 was identified as a narrowed IVDs with 23.0% (31/135) (Figures 4, 5). On the other hand, when MRI images were used to identify areas where compression of the spinal cord parenchyma was evident, T12-T13 was most frequently identified at 47.37% (9/19) and T13-L1 at 44.44% (8/18) among caudal thoracic vertebrae images, and L1-L2 was most frequently identified at 57.14% (4/7) among lumbar vertebrae images. For T10-T11 (0/13), the site of compression of the spinal cord parenchyma was not identified (Figures 4, 5).

### 3.3 Deep learning model shows good performance in detecting narrowed IVDs space sites in lateral X-ray images in a short time

When checking the interclass correlation (ICC) among veterinary clinicians for the evaluation of narrowed IVDs sites on caudal thoracic and lumbar lateral X-ray images, the kappa value was 0.812, indicating almost perfect agreement (Table 1).

**Table 1:** Interclass correlation among veterinary clinicians on the assessment of IVDs narrowing in caudal thoracic and lumbar lateral X-ray images.

	Kappa value	*p*-value	95% CI
Measure of agreement	0.812	< 0.001	0.782–0.842

CI, confidence interval.

**Figure 6:**
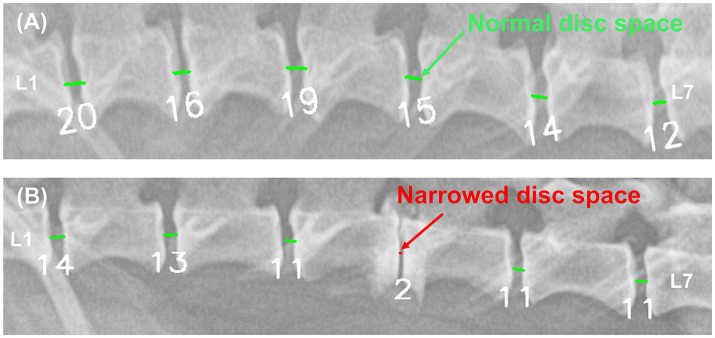
Example results from a deep learning model automatically quantifying IVDs distances and detecting areas of IVDs narrowing. In the image **(A)** of a 5.1 kg Pekingese, the deep learning model quantified the IVDs from 12–20. In the image **(B)** of a 3.8 kg Mixed breed, the deep learning model quantified the IVDs from 2–14. Each IVDs was detected as a narrow site (red) and a normal site (light green).

**Figure 7:**
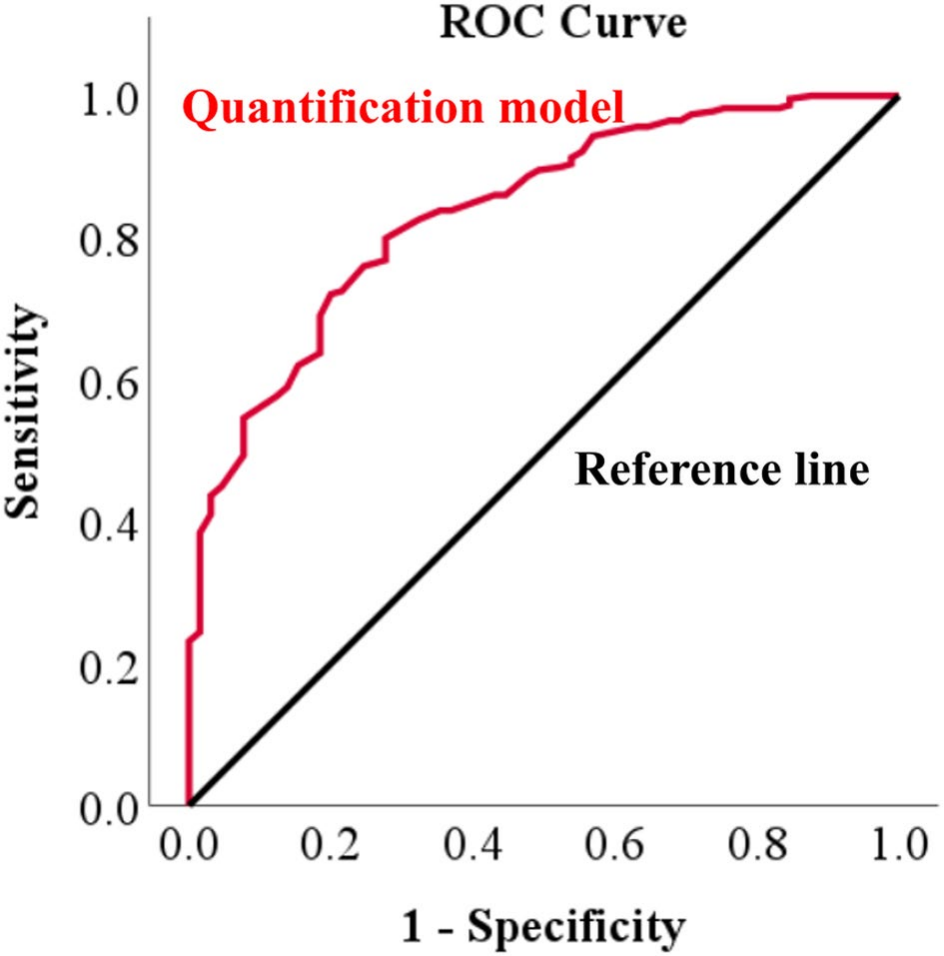
Receiver operating characteristic (ROC) curve of IVDs narrowing determined by a deep learning model versus veterinary clinician judgments in X-ray images. To evaluate how similar the IVDs narrowing determined by the deep learning model was to the narrowing determined by a veterinary clinician, ROC curve analysis was performed, identifying an area under the curve (AUC) of 0.837.

Figure 6 shows an example of a deep learning model that automatically quantifies the IVDs distance in pixels and detects narrowed IVDs sites. To evaluate the correlation between the IVDs distance quantified by deep learning and the IVDs judged as narrow by veterinary clinicians, we averaged the quantified IVDs distance, calculated the ratio of each IVDs distance to the average, and performed a ROC curve analysis between this ratio value and the IVDs judged as narrow by veterinary clinicians. The correlation between the quantified IVDs distance by deep learning and the IVDs judged narrow by the veterinary clinicians through visual assessment had an AUC of 0.837 (95% CI 0.786–0.888), indicating a very good classifier (Figure 7). When the agreement between the IVDs sites was narrowed by veterinary clinicians and the deep learning model in the validation dataset, the kappa value was 0.780, indicating substantial agreement (Table 2).

**Table 2:** Cohen’s kappa analysis between veterinary clinicians and deep learning model for detection of narrowed IVDs sites in caudal thoracic and lumbar lateral X-ray images in validation dataset.

Targeted image area	Kappa value	95% CI	Sensitivity (%)	Specificity (%)	PPV (%)	NPV (%)
Caudal thoracic	0.795^**^	0.673–0.917	87.5	93.6	82.4	95.7
Lumbar	0.763^**^	0.636–0.890	75.8	97.0	86.2	94.2
Total	0.780^**^	0.692–0.868	81.5	95.6	84.1	94.8

CI, confidence interval; PPV, positive predictive value; NPV, negative predictive value. Experimental values were considered significant at *p < 0.05 or **p < 0.01.

The mean time taken by deep learning to automatically quantify the IVDs distance and detect narrowed IVDs sites was 0.104 s per image using an RTX A6000 48GB graphics processing unit (Nvidia Corp., Santa Clara, CA, USA), whereas the evaluation by a veterinary clinician took 12.2 s per image.

### 3.4 Correlation between narrowed IVDs sites on caudal thoracic and lumbar lateral X-ray images and IVDs narrowing and spinal cord compression sites on MR images

When confirming the ICC between veterinary clinicians for the assessment of IVDs narrowing and spinal cord compression in the median plane T1-weighted and T2-weighted images, the kappa values were 0.621 and 0.784, respectively, indicating substantial agreement (Table 3).

**Table 3:** Interclass correlation among veterinary clinicians on the assessment of IVDs narrowing and spinal compression in caudal thoracic and lumbar MR images.

	Kappa value	*P*-value	95% CI
Intervertebral disc space narrowing	0.621	< 0.001	0.494–0.748
Spinal cord compression	0.784	< 0.001	0.674–0.894

CI, confidence interval; IVDs, Intervertebral disc space.

Based on the areas judged as IVDs narrowing by veterinary clinicians in the median plane T1-weighted and T2-weighted images, the association with the areas considered narrow by both veterinary clinicians and the deep learning model in the X-ray images yielded a kappa value of 0.527 for veterinary clinicians and 0.468 for the deep learning model (Table 4). Excluding the T10-T11 and L5-L6 regions showed moderate agreement, with a kappa value of 0.585 for clinicians and 0.511 for the deep learning model (Table 5).

**Table 4:** Comparison of similarity of areas of IVDs narrowing and spinal cord compression on MR images to areas judged as IVDs narrowing by veterinary clinicians and a deep learning model on X-ray images in validation dataset.

MRI findings	Targeted image area	Intervertebral disc space narrowing confirmed by X-ray	Kappa value	Sensitivity (%)	Specificity (%)
Intervertebral disc space narrowing	Caudal thoracic	Clinician	0.539**	83.3	80.0
		DL	0.418*	66.7	80.0
	Lumbar	Clinician	0.516*	71.4	82.4
		DL	0.516*	71.4	82.4
	Total	Clinician	0.527^**^	76.9	81.1
		DL	0.468^**^	69.2	81.1
Spinal cord compression	Caudal thoracic	Clinician	0.391*	83.3	66.7
		DL	0.333	66.7	72.2
	Lumbar	Clinician	0.062	50.0	61.5
		DL	0.118	50.0	69.2
	Total	Clinician	0.279^*^	75.0	64.5
		DL	0.262	62.5	71.0

DL, deep learning model; CI, confidence interval; MRI, magnetic resonance imaging; IVDs, Intervertebral disc space. Experimental values were considered significant at *p < 0.05 or **p < 0.01.

**Table 5:** Comparison of similarity of areas of IVDs narrowing and spinal cord compression on MR images to areas judged as IVDs narrowing by veterinary clinicians and a deep learning model on X-ray images, excluding T10-T11 and L5-L6 in validation dataset.

MRI findings	Targeted image area	Intervertebral disc space narrowing confirmed by X-ray	Kappa value	Sensitivity (%)	Specificity (%)
Intervertebral disc space narrowing	Caudal thoracic	Clinician	0.553**	80.0	83.3
		DL	0.404	60.0	83.3
	Lumbar	Clinician	0.625**	66.7	92.9
		DL	0.625**	66.7	92.9
	Total	Clinician	0.585^**^	72.7	87.5
		DL	0.511^**^	63.6	87.5
Spinal cord compression	Caudal thoracic	Clinician	0.576**	83.3	80.0
		DL	0.533*	66.7	86.7
	Lumbar	Clinician	0.143	50.0	70.0
		DL	0.250	50.0	80.0
	Total	Clinician	0.436^**^	75.0	76.0
		DL	0.446^*^	62.5	84.0

DL, deep learning model; CI, confidence interval; MRI, magnetic resonance imaging; IVDs, Intervertebral disc space. Experimental values were considered significant at ^*^p < 0.05 or **p < 0.01.

Based on the area of suspected IVDs in which compression of the adjacent spinal cord was confirmed by the clinician in the transverse plane T1-weighted, T2-weighted, and median plane T2-weighted images, the association with the area considered narrow by the veterinary clinician and deep learning model in the X-ray images was checked. The kappa value was 0.279 for veterinary clinicians and 0.262 for the deep learning model, indicating fair agreement (Table 4). Excluding the T10-T11 and L5-L6 regions showed moderate agreement, with a kappa value of 0.436 for veterinary clinicians and 0.446 for the deep learning model (Table 5). When spinal cord compression from MRIs of all 30 dogs (not the 9 dogs in the validation dataset) was compared to areas where the veterinarian radiologically determined narrowed intervertebral IVDs, the kappa value was found to be 0.285, with the exception of T10-T11 and L5-L6, where it was 0.392, similar to the results in the validation dataset (Table 6).

**Table 6:** Comparison of similarity of areas of spinal cord compression on MR images to areas judged as IVDs narrowing by veterinary clinicians on X-ray images, with and without T10-T11 and L5-L6 in total dataset.

	Targeted image area	Kappa value	Sensitivity (%)	Specificity (%)
With T10-T11, L5-L6	Caudal thoracic	0.220*	58.3	67.6
	Lumbar	0.404**	75.0	80.0
	Total	0.285^**^	62.5	72.4
Without T10-T11, L5-L6	Caudal thoracic	0.345**	60.9	75.9
	Lumbar	0.485**	75.0	82.9
	Total	0.392**	64.5	78.5

Experimental values were considered significant at ^*^p < 0.05 or ^**^p < 0.01.

**Figure 8:**
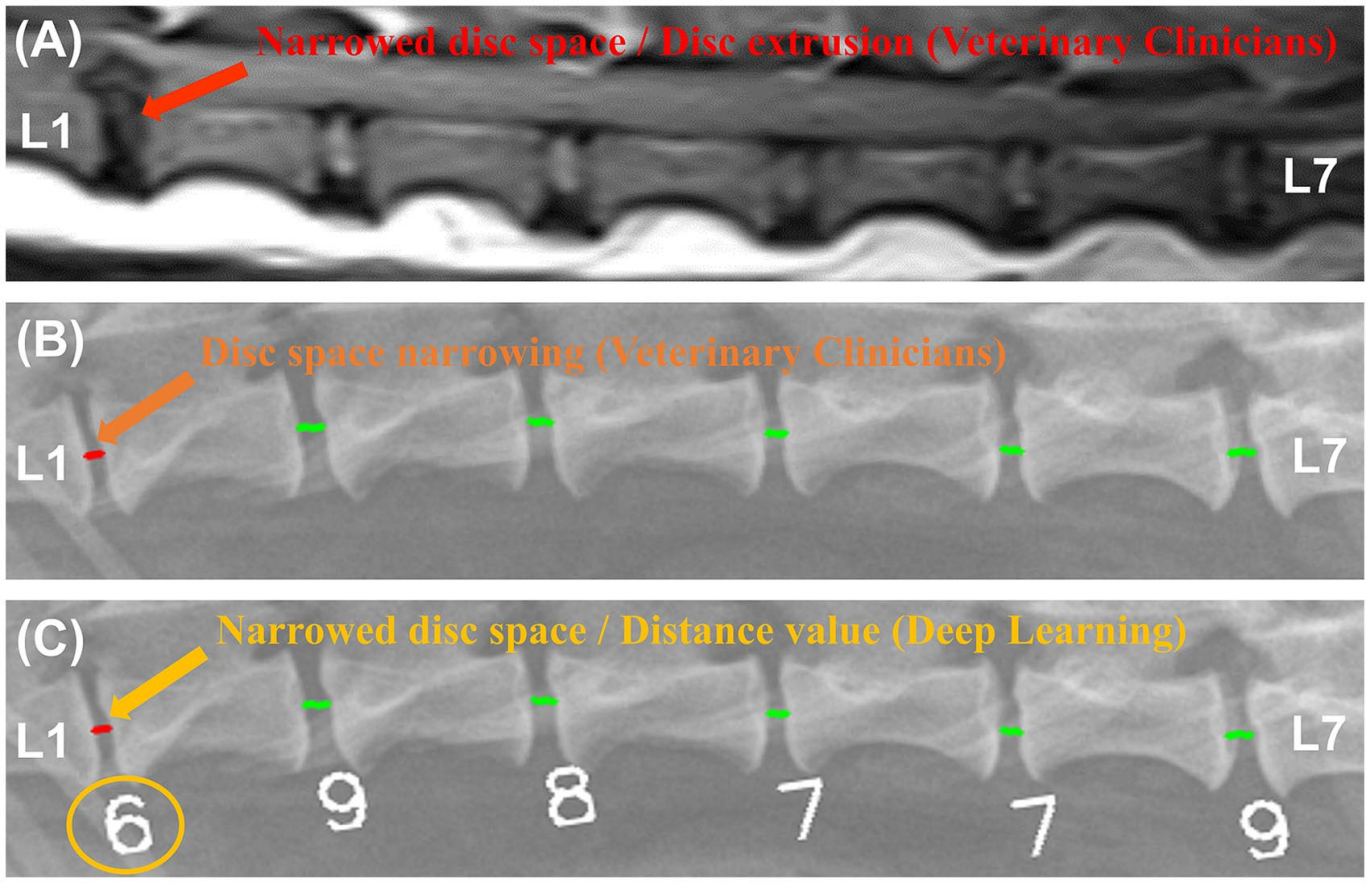
Examples of magnetic resonance imaging (MRI) and X-ray image diagnosis by veterinary clinicians and deep learning models. In a median plane T1-weighted MR image **(A)**, narrowing of the L1-L2 IVDs and spinal cord compression were identified. In an X-ray image of the same region, a veterinarian **(B)** and a deep learning model **(C)** identified the same narrowing site.

Figure 8 shows examples of a veterinary clinician’s diagnosis from MRI and X-ray images and examples of a deep learning model’s diagnosis from X-ray images.

## 4 Discussion

This is the first study to develop deep learning models to automatically quantify IVDs distance and detect narrowed IVDs in lateral caudal thoracic and lumbar X-ray images of dogs. Previous studies have shown that IVDs narrowing is the most common radiographic finding (70%) in dogs with IVDE, which causes acute ataxia due to spinal cord compression and is also a relatively common finding (25%) in IVD protrusion (IVDP) (2, 10).

A previous study developed a deep learning model to accurately segment vertebral bodies and automatically detect spondylosis deformans in radiological images of dogs, although it did not extend to automatically detecting narrowed IVDs areas due to the relatively low segmentation accuracy for IVDs (27). Therefore, this study aimed to develop a deep learning model to automatically quantify IVDs distances and detect IVDs narrowing regions based on segmented vertebral bodies.

Because IVDs distances are expressed as pixel values in the acquired images, they vary among individuals based on factors such as weight, breed, age, and X-ray acquisition conditions (especially FOV), in addition to IVDs narrowing due to disc diseases. As shown in Figure 6, a 3.8 kg Mixed dog had values ranging from 2 to 14, whereas a 5.1 kg Pekingese dog with similar body weight had relatively large values ranging from 12 to 20. While these quantified IVDs distances are useful for identifying areas of relative narrowing within an individual, the visibility to directly detect narrowing sites is somewhat limited. Adding a feature to automatically detect narrowed IVDs along with the quantified values was considered to save diagnostic time in clinical practice. Therefore, a model was developed to automatically detect narrowed IVDs by retraining to compare the quantified values to the areas that the veterinarian determined to be narrowed.

Compared to veterinary clinicians, the deep learning model for automatically detecting narrowed IVDs showed a sensitivity of 81.5% (53/65) and a very high specificity of 95.6% (219/229), resulting in an accuracy of 92.5%. This was based on correctly recognizing 272 IVDs out of 294 in the 49 X-ray images analyzed in the validation dataset, matching the assessments of the veterinary clinicians. In contrast, the sensitivity of the quantified IVDs was 72.1%, and the specificity was 80.0%, which seemed relatively low. However, with an AUC value of 0.837, it is a very good classifier, indicating high accuracy for quantification values. The difference in sensitivity and specificity values is likely due to differences in the performance evaluation methodologies used for the quantification model versus the detection model for narrowed IVDs sites.

The deep learning model is expected to be 117 times faster than veterinarians at automatically quantifying IVDs distance in pixels and detecting areas of narrowed IVDs on radiographs, significantly reducing interpretation time for veterinary clinicians. Future studies should leverage recent advancements in lightweight deep neural networks and hardware acceleration for real-time analysis on various imaging devices (28–30).

In this study, when veterinary clinicians evaluated the agreement between their identifications of narrowed IVDs sites on lateral X-ray images and median plane MR images, revealing moderate agreement with a kappa value of 0.527. Notably, the IVDs narrowing appeared somewhat underestimated on MR images, likely influenced by several factors. Firstly, the MR images were acquired under general respiratory anesthesia, which might have relaxed muscles and increased IVDs distance (31). Additionally, lateral X-ray images provide a superimposed cross-sectional view of the vertebral body, including the most cranial and caudal end plates, whereas median plane T1 and T2-weighted MR images offer a two-dimensional cross-sectional view of the IVDs, likely leading to slight underestimation of narrowing. The different directions of force applied to the vertebral body could also have influenced the findings, as radiographic images were taken from a lateral position, whereas MR images were taken from a ventro-dorsal position.

IVDs narrowing can also occur as a degenerative change associated with dehydration of nucleus pulposus cells without specific clinical symptoms related to disc disease (2, 32, 33). Typically, T10-T11 and L5-L6 discs in most dogs are observed to be narrower than adjacent IVDs under normal conditions (19). Without these physiological and anatomical considerations, the radiographic interpretation of IVDs can result in erroneous conclusions. Similar to previous studies (19), this study also showed that 61.8% (63/102) of the T10-T11 IVDs and 23.0% (31/135) of the L5-L6 IVDs were identified as narrow, representing a higher percentage compared to other adjacent IVDs (20.5%). However, no cases were found to have obvious spinal cord parenchymal compression in T10-T11 (0/13) and L5-L6 (0/10) on actual MRI images.

In the validation dataset, when comparing the areas diagnosed with IVDD due to spinal cord compression on MR images and IVDs narrowing on X-rays, veterinary clinicians and the deep learning model exhibited a sensitivity of 75.0% (6/8) and 62.5% (5/8), respectively. This sensitivity remained the same when excluding the T10-T11 and L5-L6 areas, which can appear narrow under normal conditions. However, excluding these regions led to an increase in specificity from 64.5% (20/31) to 76.0% (19/25) for veterinary clinicians and from 71.0% (22/31) to 84.0% (21/25) for the deep learning model. Moreover, in the kappa analysis, the agreement also increased from fair to moderate agreement. This indicates that many sites in the T10-T11 and L5-L6 regions appear narrow on radiographs and do not exhibit spinal cord compression.

This study also reported similar findings to previous research indicating that narrowed IVD space can diagnose IVDD in dogs with a sensitivity ranging from 64 to 69% (8). However, the results were based on MR images of only nine dogs in the validation dataset, with a kappa value below 0.5 and a positive predictive value below 60%, suggesting that assessing IVDD based solely on radiographic IVDs narrowing may be inaccurate in this study. Generally, the somewhat lower accuracy in diagnosing IVDD from radiological imaging is attributed to differences in the modalities of X-ray and MRI. X-ray serves as a screening tool focusing on vertebral alignment and IVDs narrowing due to herniation, whereas MRI is oriented toward identifying spinal cord compression and parenchymal lesions rather than observing vertebral and IVDs abnormalities. Therefore, a comprehensive approach combining radiographic findings with physical and neurological examinations, alongside MR imaging findings, is essential for diagnosing disc diseases. Further studies integrating the model developed in this study with a model for vertebral body segmentation and detection of spondylosis deformans can enhance the diagnostic utility for IVDD (27).

This study had a few limitations. First, since a slightly wider range of vertebral bodies were acquired in a single image for training the deep learning model, the assessment of IVDs in off-center regions may be somewhat inaccurate along the patient’s weight, length and depending on patient to detector distance even though the FDD was fixed at a relatively long 80 cm. In addition, even when restricted to dogs over 10 kg, the validation dataset showed above moderate agreement with kappa values above 0.55 for the caudal thoracic vertebrae and lumbar also, but the population of medium and large dogs over 10 kg was somewhat insufficient compared to the population of small dogs under 10 kg. Further training and validation of the deep learning model on a variety of different ranges of images, different body weights, and breeds will be required to address these concerns. Secondly, the small number of MR image samples available for comparison with the validation data might have led to inaccuracies in the comparisons between radiographic and MRI findings. And the use of low-field MRI in this study restricted the ability to obtain slices thinner than 2.5 mm, potentially reducing the accuracy in assessing the degree of IVDs narrowing or spinal cord compression in smaller dogs weighing 3 kg or less. Finally, this study did not consider diseases that can cause narrowing of the intervertebral IVDs without compression of the spinal cord parenchyma, such as acute non-compressive nucleus pulposus extrusion (ANNPE), which was not included in the dogs who underwent MRI. Further studies with larger sample sizes, including high-field MR scans, are required to overcome these limitations. Furthermore, by utilizing high-field MRI, it is thought that it will be possible to develop a model that can detect not only IVDs narrowing in radiographic images, but also degeneration in the IVDs.

In conclusion, this study successfully developed a deep learning model capable of automatically quantifying IVDs distance on a pixel basis and detecting narrowed IVDs sites in caudal thoracic and lumbar lateral vertebral X-ray images of dogs. This model holds promise for screening for disc disease and facilitating rapid lesion localization. These artificial intelligence-assisted diagnostic techniques should be utilized primarily to support diagnosis and reduce the risk of missed findings, while continued advancement in diagnostic skills by practicing veterinarians remains essential. Further investigations involving diverse samples with different breeds are warranted to further validate and enhance the utility of the model.

## Data Availability

The original contributions presented in the study are included in the article/supplementary material, further inquiries can be directed to the corresponding author.
